# Fluticasone Furoate-Oxymetazoline Hydrochloride Combination Nasal Spray for Managing Allergic Rhinitis With Nasal Congestion: Current Evidence and Clinical Implications

**DOI:** 10.7759/cureus.108413

**Published:** 2026-05-07

**Authors:** Sarika Verma, K. R Meghanadh, Samit Lal, Ashwin Kotamkar, Shailesh Pallewar, Amit Qamra

**Affiliations:** 1 Allergy, AllergyDoc Clinics, Moolchand Hospital, New Delhi, IND; 2 Otolaryngology, MAA ENT Hospitals, Hyderabad, IND; 3 Otolaryngology, Dr. Lal's Hospital and Research Centre, Ranchi, IND; 4 Medical Affairs, Macleods Pharmaceuticals Ltd, Mumbai, IND

**Keywords:** allergic rhinitis, fluticasone furoate, nasal decongestants, nasal sprays, oxymetazoline, rhinitis medicamentosa

## Abstract

Intranasal corticosteroids (INCS) are the primary treatment for allergic rhinitis (AR) owing to their anti-inflammatory effects. Fluticasone furoate (FF), an INCS, is widely used because of its high glucocorticoid receptor affinity and once-daily dosing. Despite adequate INCS therapy, nasal congestion often remains the predominant symptom of moderate-to-severe AR. Guidelines recommend the short-term use of nasal decongestants as adjunctive therapy for AR with significant nasal congestion. Oxymetazoline (OXY), a long-acting α-adrenergic agonist, relieves nasal congestion by vasoconstriction. It starts working within 25 seconds and lasts approximately 10-12 hours. However, its use as monotherapy is limited to three to five days to avoid rhinitis medicamentosa (RM), a condition characterized by increased nasal congestion resulting from extended use of this medication. The combination of an INCS (FF) and a nasal decongestant (OXY) may provide a synergistic approach, with FF targeting inflammation and OXY providing rapid symptomatic relief from nasal congestion. This review summarizes the efficacy and safety of the FF+OXY fixed-dose combination (FDC) nasal spray, with a focus on pharmacological synergy. A literature review using PubMed and Google Scholar identified studies on the FF+OXY (27.5/50 µg) FDC nasal spray. Clinical evidence has shown that short-term use (≤14 days) of the FF+OXY (27.5/50 µg) FDC nasal spray provides rapid and sustained relief without a significant risk of RM. While few clinical trials have demonstrated the potential efficacy and safety of this FDC, there is a need for additional clinical trials and real-world evidence, given the widespread use of the FF+OXY (27.5/50 µg) FDC nasal spray for managing moderate to severe AR with nasal congestion in India.

## Introduction and background

Allergic rhinitis (AR) overview and global burden

Allergic disorders, such as allergic rhinitis (AR), asthma, atopic dermatitis, and food allergies, result from immune system dysfunction. The increasing incidence and recurrence rates of allergic disorders, including AR, have garnered attention. The World Health Organization (WHO) has listed allergic diseases among the top three disorders that require prevention in the 21st century [1,2]. AR is an immunoglobulin E (IgE)-mediated reaction to inhaled allergens, characterized by inflammation of the nasal mucosa and nasal passages, nasal discharge, obstruction, sneezing, or itching, with symptoms lasting at least one hour daily for many days (including two consecutive days) [3]. The "Allergic Rhinitis and its Impact on Asthma" (ARIA) initiative classifies AR as intermittent/persistent and mild or moderate/severe, with diagnosis based on clinical history, skin tests, or detection of serum-specific IgE [4]. AR is a common chronic condition in high-income countries, with a prevalence of up to 50% [2]. Globally, the prevalence of AR continues to rise, particularly in low- and middle-income countries, including India, ranging from 1.0% to 54.5% [2,5].

AR burden in India

India is home to nearly 20% of the global population, with approximately 1.35 billion people. The incidence of allergic diseases, such as AR and asthma, has recently increased in India. Approximately 22% of Indian adolescents have AR, although this may not reflect the actual burden due to limited epidemiological studies, especially in rural and semi-urban areas [6,7]. A nationwide physician survey by Gupte et al. reported an AR prevalence of 21-40% in clinical practice [8]. While AR risk factors are primarily environmental or genetic, new factors such as proximity to dumpsters, vehicle movement, and nighttime exposure to artificial light have been associated with AR development. However, AR diagnosis and management in India remain suboptimal owing to limited allergy training, diagnostic facilities, and high drug costs [6,7].

Classification of AR

AR is classified by exposure pattern: seasonal, perennial, or episodic (e.g., pets). Symptoms frequency: Intermittent (<4 days/week or <4 weeks/year) or persistent (>4 days/week and >4 weeks/year). Severity: Mild (no quality of life (QoL) interference) or moderate-to-severe (QoL interference). An Indian expert consensus suggests further subgrouping intermittent/persistent or mild/moderate/severe AR into “sneeze-runners” (sneezing, rhinorrhea, itchy nose/eyes; pollen sensitization) and “blockers” (nasal blockage, thick mucus, postnasal drip, breathlessness; sensitization to house dust, mites, fungi) due to distinct profiles and treatments. Symptoms may overlap as “combined type.” “Blockers” often have persistent AR, while “sneeze-runners” have intermittent AR [9].

Understanding pathophysiology

Allergic responses to AR involve early and late phases of the disease. The early phase begins within 20 min of allergen exposure. Dendritic cells process allergen peptides on major histocompatibility complex (MHC) class II molecules, thereby activating naïve cluster of differentiation (CD4+) T cells into Th2 cells. Interleukins (IL-4 and IL-13) trigger B lymphocytes (B cells) to produce allergen-specific IgE, which binds to the high-affinity IgE receptor (FcεR) on mast cells [10-12]. FcεR cross-linking releases mediators, such as histamine and proteases, which cause inflammation [10,13-15]. These mediators induce mucosal edema and rhinorrhea in the nasal cavity. Interactions between histamine and H1 receptors cause sneezing and nasal congestion. The late phase begins four to six hours after exposure, when inflammatory cells infiltrate the nasal mucosa. IL-4 and IL-5 trigger this process by upregulating vascular cell adhesion molecule (VCAM-1) expression in endothelial cells [10,16].

Treatment of AR

Allergen avoidance and pharmacotherapy are crucial for the management of AR [17]. Pharmacological options include antihistamines, intranasal corticosteroids (INCS), leukotriene receptor antagonists (LTRAs), and immunotherapy [18]. INCS is recommended as a first-line therapy for patients with moderate-to-severe AR, especially when nasal congestion is a significant symptom [19]. Approved INCS include mometasone furoate (in patients aged ≥2 years), fluticasone propionate (in patients aged ≥4 years), fluticasone furoate (FF) (in patients aged ≥2 years), triamcinolone acetonide (in patients aged ≥4 years), and ciclesonide (in patients aged ≥6 years) [20]. First-generation antihistamines include diphenhydramine and chlorpheniramine, whereas fexofenadine, loratadine, and cetirizine are second-generation antihistamines. Although both generations effectively control AR symptoms, LTRAs such as montelukast are less effective than INCS. Oral decongestants, such as pseudoephedrine, relieve symptoms but are not recommended for extended use. Intranasal decongestants, such as xylometazoline and oxymetazoline (OXY), are alpha-agonists that induce vasoconstriction in the nasal tissue [18].

Pharmacotherapy: Monotherapy

An Indian expert consensus recommends INCS monotherapy for initial nasal AR symptoms in patients aged ≥12 years. INCSs are effective for nasal and ocular symptoms and sleep disturbance, with adjustments if sleep issues are due to postnasal drip, congestion, itching, or sneezing. INCS spray is more effective than an antihistamine spray for AR symptoms. Intranasal fluticasone furoate (FF) 110 μg daily is preferred for consistent efficacy and patient satisfaction in AR, with favorable sensory attributes. Oral second-generation antihistamines may be prescribed for "sneeze-runners" or for mild-to-moderate seasonal and mild perennial AR. Intranasal antihistamines are recommended for "sneeze-runners" and episodic AR, offering a faster onset and fewer side effects than oral antihistamines. LTRA is not recommended for initial AR treatment but may improve symptoms in AR patients with asthma. Isotonic intranasal saline is recommended for all AR patients, with buffered and non-buffered options available for adults and children [9].

Limitations of monotherapy

Clinical evidence suggests that approximately 40% of patients with AR do not achieve complete symptom relief with INCS alone, indicating the need for enhanced or adjunctive treatment approaches [21]. Wang et al. concluded that some patients with chronic rhinitis still experience persistent nasal congestion despite adequate INCS treatment [22]. Nasal congestion is one of the most vexing symptoms of AR. INCSs can relieve nasal congestion, but their effects are not immediate and may take several days to become evident. This may lead to discontinuation of therapy or noncompliance by patients [9]. Nasal decongestants, often used for rapid relief of nasal congestion, are readily available over-the-counter (OTC). Additionally, chronic use of intranasal decongestants results in tachyphylaxis and rebound congestion, also known as rhinitis medicamentosa (RM), which is attributed to α-adrenoceptor-mediated downregulation and desensitization [23]. As nasal congestion severely affects the quality of life (QoL) of patients with AR, additional medications and combined therapies are needed for moderate-to-severe cases until intranasal steroids reach full efficacy [21]. Clinicians should review the etiology of nasal sinus disease in patients with persistent congestion despite the use of nasal decongestants or INCS or worsening after discontinuation. Combination therapy and surgical treatment have also been proposed [23].

Pharmacotherapy: Combination therapy

An Indian expert consensus recommends combination pharmacological therapy for moderate/severe persistent AR and for inadequate response to monotherapy. The experts recommend daily INCS and intranasal decongestants for faster, greater reduction in nasal congestion, improved nasal volume, and prevention of rhinitis medicamentosa from prolonged decongestant use, achieved by limiting decongestant use to once daily for the “blockers” phenotype and for moderate-to-severe seasonal or perennial AR. They recommend evening fluticasone furoate (FF) and oxymetazoline (OXY) hydrochloride (HCL) nasal spray, 27.5/50 mcg, for moderate-to-severe AR patients with nasal congestion without significant risk of RM [9].

Various other guidelines recommend intranasal corticosteroids (INCS) and nasal decongestants for the management of AR

The 2024-2025 ARIA guidelines recommend intranasal decongestants (oxymetazoline, xylometazoline, and tramazoline) for AR patients due to their over-the-counter availability and rapid onset of action. However, they recommend against long-term use (over five days) compared to no treatment [24]. The Joint Task Force on Practice Parameters in Allergy, Asthma, and Immunology of the American Academy of Allergy, Asthma, and Immunology (AAAAI, 1998) and the American College of Allergy, Asthma, and Immunology (ACAAI-2000) consider INCS as the first-line therapy when nasal congestion is a principal component of the patient’s presentation [25,26]. The guidelines from the European Forum for Research and Education in Allergy and Airway Diseases (EUFOREA) recommend intranasal decongestants as an add-on therapy for ≤7 days for patients with AR [27]. The International Consensus Statement on Allergy and Rhinology: Allergic Rhinitis 2023 states that “Intranasal decongestants can provide effective short-term relief of nasal congestion in patients with AR during an acute flare, but recommend against chronic use due to the risk of RM [28]." The Ministry of Health in Singapore recommends the use of topical (intranasal) decongestants for up to three days in adolescents and adults [29]. The National Health Systems Resource Center (NHSRC) in India recommends the use of topical decongestants for a maximum of five days to manage severe nasal congestion [30]. While these guidelines highlight the value of both INCS and intranasal decongestants in AR management, they also highlight a crucial therapeutic gap. Monotherapy with either approach has limitations and necessitates adjunctive or combination strategies when indicated.

Need for combination pharmacotherapies-focus on fixed-dose combination (FDC) nasal spray of fluticasone furoate (FF) (27.5 µg)+oxymetazoline (OXY) (50 µg)

Given the limitations of monotherapy, attention has turned to combination pharmacotherapies. Evidence demonstrates that intranasal decongestants, when combined with INCS and administered for four weeks, significantly improved nasal symptom scores compared with monotherapy with either agent. A combination of intranasal decongestants with INCS is recommended for AR and severe nasal congestion, demonstrating synergistic effects in reducing congestion and runny nose without causing drug-induced rhinitis [31,32]. The “clinical practice guidelines published in Otolaryngology-Head and Neck Surgery” (American Academy of Otolaryngology-Head and Neck Surgery Foundation (AAO-HNSF), 2015), comprising 20 experts across specialties, recommend promoting effective pharmacological combinations and reducing ineffective ones for AR treatment. Although oral antihistamines and INCS are common monotherapies, their combination provides limited benefit in the treatment of AR [33].

Greiwe et al. highlighted that individualized combination therapies, including INCS and intranasal decongestants, are effective in managing complex AR phenotypes and improving patient outcomes [34]. Neighbors et al. systematically reviewed the evidence for the combination treatment of INCS spray with OXY nasal spray for chronic rhinitis. The results showed a greater improvement in nasal congestion in the combined treatment group than in the control group. A combination treatment demonstrated that a higher nasal volume was more effective in reducing rhinitis symptoms than either treatment alone, without causing RM [35]. Vaidyanathan et al. demonstrated that OXY-induced tachyphylaxis and rebound congestion could be reversed by combining OXY with intranasal fluticasone in patients with AR [36]. A meta-analysis by Chitsuthipakorn et al. included four studies comparing INCS decongestion with INCS alone, demonstrating that the combination of INCS and nasal decongestants prevents rebound congestion [37]. However, further evidence is needed to assess whether the combination of OXY and FF is an effective short-term strategy for alleviating tachyphylaxis, rebound congestion, and RM in patients with AR.

Despite the wide availability of pharmacotherapy for AR, nasal congestion remains a bothersome symptom. Intranasal decongestants, such as OXY, are frequently used in clinical practice for symptomatic relief; however, prolonged use is associated with RM. In contrast, INCS, such as FF, provide sustained anti-inflammatory benefits but have a slower onset of action. Although the efficacy of FF and OXY as monotherapies for managing AR is well documented, research on the use of FF+OXY (27.5/50 µg) fixed-dose combination (FDC) nasal spray without the development of RM remains limited. This narrative review synthesizes current evidence on the short-term effectiveness and safety of FF+OXY (27.5/50 µg) for treating moderate-to-severe allergic rhinitis (AR) characterized by nasal congestion.

## Review

Literature search strategy

A literature search using PubMed and Google Scholar identified studies on FF and OXY as monotherapy and in combination for moderate-to-severe AR with nasal congestion management. The search used keywords and Boolean operators, including (“fluticasone furoate” AND “oxymetazoline”) AND (“allergic rhinitis” OR “AR” OR “seasonal allergic rhinitis” OR “SAR” OR “perennial allergic rhinitis” OR “PAR”) AND (“nasal congestion” OR “nasal obstruction”) AND (“moderate” OR “severe”). Filters for human studies and the English language were applied. Studies focused on efficacy, safety, and clinical use of FF+OXY in moderate-to-severe AR with nasal congestion.

Pharmacologic overview of the FF+OXY (27.5/50 µg) FDC nasal spray (active ingredients)

Table 1 presents the pharmacological profiles of FF and OXY for the management of AR. FF, due to its high potency and prolonged nasal retention, provides sustained anti-inflammatory effects for daily use. Similarly, OXY provides rapid symptomatic relief through vasoconstriction, with an onset of action of 25 seconds, rapidly reducing nasal congestion. However, its use as monotherapy is recommended for only three to five days due to the risk of RM. This combination utilizes the long-term control of inflammation by FF combined with the rapid decongestant effect of OXY, thus providing a synergistic approach for managing inflammation and nasal congestion in patients with AR. The risk of rebound congestion with prolonged OXY use necessitates appropriate duration and dosing strategies for FDC nasal sprays. When OXY is administered with an INCS, the duration of action of the former increases, demonstrating good efficacy even with once-daily dosing; the effect persists throughout the day, improving QoL. Mild AEs included nasal/mouth dryness and bitter taste. This pharmacological synergy supports the use of FF+OXY (27.5/50 µg) FDC nasal spray in patients with moderate-to-severe AR [27-58].

**Table 1:** Pharmacological profile of FF and OXY FF: fluticasone furoate, OXY: oxymetazoline, OTC: over the counter, RM: rhinitis medicamentosa, QoL: quality of life. Table recreated from Refs. [27-58].

Characteristics	Fluticasone furoate (27.5 µg)	Oxymetazoline (50 µg)
Class	Intranasal corticosteroid	Nasal decongestant (α-adrenergic agonist)
Mechanism of action	Anti-inflammatory action via glucocorticoid receptor activation	Non-selective α1 and α2 adrenergic receptor agonist causing vasoconstriction
Onset of action	Gradual, sustained over time	Rapid: within ~25 seconds
Duration of action	~24 hours	~10–12 hours
Therapeutic benefits	Relief of nasal and ocular symptoms, improves the quality of life	Rapid relief of nasal congestion, mild relief of sneezing, rhinorrhoea, and nasal itching
Risks/limitations	Minimal side effects	Risk of rhinitis medicamentosa, mucosal alterations
Regulatory status	Prescription medication	OTC medication

FF+OXY FDC: Therapeutic rationale and formulation overview

FF+OXY (27.5/50 µg) FDC nasal spray presents a synergistic strategy for managing moderate-to-severe AR patients with nasal congestion. FF, first approved in the United States of America (USA) in 2007, is a potent trifluorinated INCS that exhibits anti-inflammatory properties by inhibiting inflammatory mediators and reducing mucosal edema. Its onset was gradual, with optimal effects observed over a few days. OXY, first approved in the USA in 1975, is an adrenergic α1- and α2-agonist, and a direct-acting sympathomimetic drug. By stimulating adrenergic receptors, OXY causes vasoconstriction of dilated arterioles, with an onset of action in 25 seconds, thereby reducing the blood flow through these vessels. When sprayed intranasally, OXY relieved nasal congestion and improved nasal airflow in patients with AR for up to 12 hours following a single dose. OXY relieves nasal congestion by vasoconstricting respiratory microvessels, including both resistance and capacitance vessels in the human nasal mucosa, thereby reducing nasal mucosal blood flow, edema, and airflow resistance.

**Figure 1:**
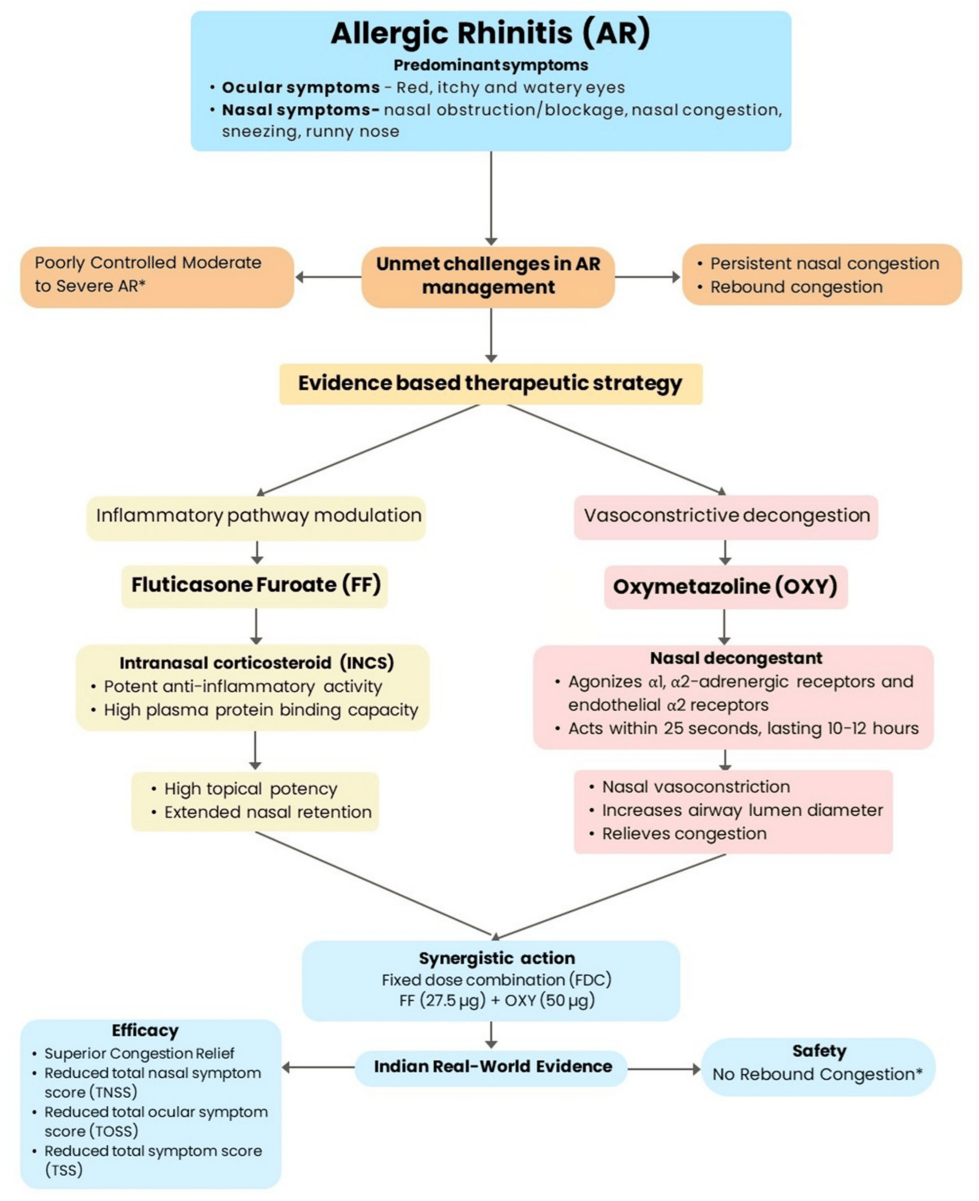
FF+OXY (27.5/50 µg) FDC nasal spray-therapeutic rationale in managing AR. *Monotherapy with INCS/nasal decongestants Figure recreated from Refs. [27-58]. AR: allergic rhinitis; FF: fluticasone furoate; OXY: oxymetazoline; INCS: intranasal corticosteroid.

When administered with FF, OXY provided immediate relief by opening the nasal passages and facilitating better penetration of FF's anti-inflammatory effects. FF ensures long-term control by addressing the underlying inflammatory aspects of AR. This dual-action approach enhances patient comfort and improves treatment efficacy. FF may reduce rebound congestion associated with OXY by stabilizing mucosal inflammation. Thus, the FF+OXY (27.5/50 µg) FDC nasal spray targets both the cause and consequence of nasal congestion, providing a practical option for managing AR [27-58] (Figure 1). The FDC nasal spray of FF (micronized) and OXY HCL was approved in India in July 2021. Each spray delivers 27.5 µg of FF and 50 µg of OXY.

FF as monotherapy in the management of AR-existing evidence

Evidence from randomized trials and real-world surveys highlights that FF effectively relieves AR symptoms compared with placebo and other INCS. Additionally, both physicians and patients report a preference for FF, suggesting greater adherence potential and alignment in treatment choices [8,46-50] (Table 2).

**Table 2:** Existing evidence supporting FF and OXY as monotherapy in the management of AR. AR: allergic rhinitis, FF: fluticasone furoate, OXY: oxymetazoline, OTC: over the counter, RM: rhinitis medicamentosa, INCS: intranasal corticosteroid, FFNS: fluticasone furoate nasal spray, FPNS: fluticasone propionate nasal spray, MFNS: mometasone furoate nasal spray, rTNSS/rTNSS: reflective/instantaneous total nasal symptom score; rTOSS/iTOSS: reflective/instantaneous total ocular symptom score; QoL: quality of life; RCT: randomized controlled trial; AEs: Adverse events; HCP: healthcare providers.

Clinical evidence supporting FF as monotherapy for managing AR					
Author	Study population (n)	Study design	Intervention	Outcomes assessed	Key findings
Rodrigo et al. [46]	5,348	Systematic review and meta-analysis	Intranasal FF versus placebo on ocular and nasal symptoms in patients with AR	rTOSS, iTOSS, rTNSS, iTNSS, QoL, AEs	Intranasal FF significantly improved rTOSS and iTOSS scores compared with placebo (weighted mean difference (WMD) -0.54, 95% CI: -0.70 to -0.37, and -0.59, 95% CI: -0.76 to -0.43) and perennial AR (-0.33, 95% CI: -0.31 to -0.05, and -0.38, 95% CI: -0.69 to -0.07). Intranasal FF was also significantly more effective in improving rTNSS and iTNSS scores in seasonal and perennial AR patients
May et al. [47]	~2,400	Narrative literature review	FFNS versus placebo or other INCS for efficacy, safety, and/or preference	Product characteristics, sensory attributes, adherence, preference	FFNS was preferred over FPNS and MFNS due to its better scent, taste, and lower drip. Improved adherence.
Gupte et al. [8]	1,608 Physicians	Cross-sectional survey	Physician preferences	Preferred INCS in moderate-to-severe AR	FF was the preferred INCS option (67.1% of physicians). The most recommended duration of INCS therapy was two to four months (40.9% of physicians).
Prabhat et al. [49]	300 Physicians	Cross-sectional survey	Preferred INCS	Preferred INCS in clinical practice	55% of physicians selected FF as the INCS of choice
Yanez et al. [50]	300 Patients	Patient preference survey	Preferred INCS	Patient preference for FFNS/MFNS based on their sensory attributes	56% of patients stated a preference for FFNS versus 32% for MFNS (p<0.001)
Clinical evidence supporting OXY as monotherapy for managing AR					
Author	Study population (n)	Study design	Intervention	Outcomes assessed	Key findings
Reinecke and Tschaikin [44]	247	A placebo-controlled double-blind study	OXY/saline spray over 10 days	The primary measure was AR duration, with onset and symptoms as secondary measures. Safety was assessed based on patient satisfaction, heart rate, and side effects.	AR duration decreased under OXY compared to the control (4 vs. 6 days). The onset of action for OXY was 25 seconds, compared to 90 seconds for saline (P<0.001). Symptoms improved significantly in the OXY group from day two onwards. Physicians rated OXY efficacy good/very good in 85% versus 38% for saline (P<0.001). 84% of OXY patients rated the effects as good/very good, compared to 44% in the saline group (P<0.001). OXY proved superior to saline solution, shortening AR duration by two days.
Watanabe et al. [51]	30	Randomized double-blind placebo-controlled trial	0.05% OXY nasal spray, two sprays (0.1 ml/spray) to each nostril three times daily over an extended period of four weeks	Nasal blockage, airflow, and nasal patency were assessed before and after four weeks of treatment, and two weeks following discontinuation of treatment.	A highly significant decongestant effect of OXY was observed at each clinic visit, with changes in all three measurements. No rebound congestion or increased blockage after four weeks.
Druce et al. [52]	128 (Two pooled RCTs)	Two randomized, double-blind, vehicle-controlled, single-dose, parallel, clinical studies	0.05% OXY spray	Nasal congestion, nasal flow, AEs	Improved nasal airflow at all time points (1–12h). Significantly better congestion scores than placebo. OXY provides both statistically significant and clinically meaningful relief of nasal congestion and improved nasal airflow for up to 12 hours following a single dose.
Scheire et al. [53]	22	Qualitative interviews	Chronic nasal decongestant overuse (≥6 months)	Patient perception, dependence, impact on QoL	Interviewees reported that nasal congestion severely impacted daily life, with only decongestants providing relief despite known risks.

OXY as monotherapy in the management of AR-existing evidence

Evidence from clinical studies has demonstrated that OXY provides rapid and significant relief from nasal congestion, with onset within 25 seconds and efficacy lasting up to 12 hours. Patient-reported outcomes highlighted the perceived necessity of OXY for daily functioning, reflecting high satisfaction and reliance on OXY for symptomatic relief. Clinical and patient-reported outcome data highlight the perceived necessity of OXY due to its decongestant action and immediate relief of nasal obstruction [43-45,51-53] (Table 2).

FF+OXY FDC in the management of AR-existing evidence

Matreja et al. conducted a randomized, open, parallel study at the outpatient department of Gian Sagar Medical College and Hospital, Patiala District, India. One hundred and twenty-three patients were randomly assigned to receive either fluticasone with oxymetazoline or fluticasone alone. Ninety-one patients completed the four-week study period. Patients were assigned to receive either fluticasone with OXY (Group 1) or fluticasone alone (Group 2). The primary outcome was the mean change in the total daytime nasal symptom score (TDTS), and the secondary outcomes were the nighttime nasal symptom score (NTS) and the composite symptom score (CSS). Changes in the daytime, composite, and nighttime nasal symptom scores were significantly (p<0.05) greater in Group 1 than in Group 2. Subgroup analysis revealed a greater improvement in the congestion score from the second week in Group 1 (p<0.05). The authors concluded that OXY combined with fluticasone was effective in reducing daytime, nighttime, and composite symptom scores compared with fluticasone alone [54].

Kumar et al. conducted a prospective, randomized, double-blind, two-arm, active-controlled, parallel, multicenter, comparative clinical study in India. This study included patients with AR aged ≥18 years with moderate-to-severe nasal congestion. A total of 250 patients were randomized (1:1) to receive either FDC (FF+OXY, 27.5/50 µg) nasal spray or FF alone, administered as two sprays in each nostril every night. FDC showed a significantly greater reduction (p<0.001) in nighttime TNSS than in FF at all time points from day 3 to day 28. A significantly higher number of patients achieved complete relief of nasal congestion with FDC (44.7%) than with FF (26.8%) (p<0.05). Both medications were well-tolerated by the patients. Post-treatment symptom worsening was similar in both groups (p>0.05). The FDC demonstrated superior efficacy compared to FF in relieving nasal congestion and reducing TNSS. The daily use of OXY combined with INCS resulted in no rebound congestion even after 28 days of continuous treatment [55].

Juvekar et al. conducted a prospective, open-label, single-arm, multicenter, real-world observational study of Indian patients with AR for 28 days. Patients were treated with FF+OXY nasal spray. The TNSS, TOSS, and total symptom score (TSS) were recorded at baseline and at the end of the study. The investigators rated treatment effectiveness on a four-point Likert scale. FF+OXY treatment showed a significant reduction in TNSS from 7.18 ± 3.38 to 0.20 ± 0.84 (p<0.001), TOSS from 2.34 ± 2.29 to 0.09 ± 0.53 (p<0.001), and TSS from 9.51 ± 4.94 to 0.29 ± 1.32 (p<0.001) after 28 days. Investigators reported excellent effectiveness in 52.12% of the patients. FF+OXY nasal spray effectively relieved nasal congestion and reduced symptoms in patients with AR. Serious adverse events were not observed (Table 3). Thus, the authors concluded that this combination is safe and does not cause rebound congestion during treatment [56].

**Table 3:** Clinical studies evaluating the efficacy and safety of FF+OXY FDC in patients with moderate-to-severe AR with nasal congestion. AR: allergic rhinitis, FF: fluticasone furoate, OXY: oxymetazoline, TNSS: total nasal symptom score; TOSS:  total ocular symptom score, TSS: total symptom score, NTS: nighttime nasal symptom score, CSS: composite symptom score.

Study	Study design	Population	Intervention	Duration	Outcomes assessed	Key findings
Matreja et al. [54]	Randomized, open-label, parallel	AR patients (n=123; 91 completed)	Group 1: FF+OXY or Group 2: FF	Four weeks	TDTS, NTS, CSS	Greater reduction in daytime, nighttime, and composite scores in Group 1 (p<0.05); significant improvement in congestion from week two in Group 1 (p<0.05).
Kumar et al. [55]	Prospective, randomized, double-blind, multicenter	Adults ≥18 years with moderate-severe AR (n=250)	FF+OXY (27.5/50 µg) or FF alone	28 days	TNSS, nasal congestion relief	Significant reduction in TNSS from day three onwards (p<0.001); Higher complete congestion relief (44.7% vs 26.8%, p<0.05); No rebound congestion observed
Juvekar et al. [56]	Prospective, open-label, single-arm, real-world	AR patients	FF+OXY nasal spray	28 days	TNSS, TOSS, TSS	Significant reduction in TNSS, TOSS, TSS (p<0.001); Investigators reported excellent effectiveness in 52.12% of the patients; No serious adverse events or rebound congestion

Strategies to optimize AR management-INCS+nasal decongestant combinations

Kirtsreesakul et al. evaluated the effects of OXY and INCS on rebound congestion. Sixty-eight patients with nasal polyposis received OXY plus mometasone furoate nasal spray (MFNS) or placebo plus MFNS, administered as two sprays per nostril twice daily for four weeks. All patients were prescribed MFNS for two weeks. Outcomes were measured using TNSS, peak inspiratory flow index, nasal mucociliary clearance time (NMCCT), and total nasal polyp scores (TNPS). At four weeks, the OXY-MFNS group demonstrated greater improvements in nasal blockage, hyposmia, peak flow, NMCCT, and polyp scores than did the placebo group. During the nasal steroid phase, both improved; however, OXY-MFNS showed greater improvement than OXY-normal saline (NS). INCS with OXY was more effective than INCS alone in improving the symptoms and polyp size. No rebound congestion occurred after treatment [57].

Expert opinion/consensus, reviews, and guidelines recommending FF+OXY FDC

The “Joint Task Force Practice Parameter on Rhinitis” (AAAAI and ACAAI, 2020) recommends that combination therapy involving INCS and nasal decongestants be administered for up to four weeks in patients experiencing nasal congestion who do not respond to INCS alone or in conjunction with intranasal antihistamine therapy [58]. Conversely, the AAO-HNSF 2015 “Clinical Practice Guideline for AR” advises restricting combination therapy with nasal decongestants to a few days to prevent rebound congestion [33]. The International Consensus Statement on Allergy and Rhinology (American Rhinology Society, United States, 2023) recommends that short-term combination therapy with INCS and nasal decongestants should be considered for patients with AR who are unresponsive to INCS and intranasal antihistamines and for those who decline surgical intervention [28]. Thus, FF+OXY (27.5/50 µg) FDC nasal spray can be considered a targeted approach for managing AR, particularly in patients with nasal congestion (Table 4).

**Table 4:** Practical considerations for using FF+OXY FDC in AR. AR: allergic rhinitis; FF: fluticasone furoate; FDC: fixed-dose combination; OXY: oxymetazoline; INCS: intranasal corticosteroid; RM: rhinitis medicamentosa. Data on “Effect on RM” is adapted from Kameswaran et al. [9].

Aspects	Key points for clinical practice
When to use	Moderate-to-severe AR with nasal congestion as a predominant or most distressing symptom. Acute symptom relief: When a patient with AR requires immediate relief from blockage (OXY) while starting long-term inflammatory control (FF). Step-up therapy: For patients with AR whose symptoms are not adequately controlled by INCS monotherapy alone.
Duration of therapy	Short term use (≤14 days or ≤2 weeks)
Effect on RM	While OXY monotherapy is restricted to a three-to-five-day window, combining it with an INCS (like fluticasone furoate (FF)) facilitates extended treatment for up to four weeks without significant risk of rhinitis medicamentosa (RM)

Strengths and limitations

To the best of our knowledge, this is the first review to address the use of the FDC nasal spray FF+OXY (27.5/50 µg) in the management of AR with nasal congestion in the Indian context. This review provides a comprehensive synthesis of the current evidence, expert opinions, guideline updates, and real-world clinical perspectives on the potential role of combining INCS with nasal decongestants, specifically the FDC nasal spray FF+OXY (27.5/50 µg), in the management of AR. It highlights the pharmacological combined effect, rapid onset of action, and clinical utility of this FDC nasal spray in the management of AR and related conditions.

Nonetheless, the review has several limitations. Firstly, as a narrative review, it did not follow a formal systematic methodology. Secondly, the evidence on the FF+OXY FDC is limited in both quantity and scope. The included studies are few and vary in design (randomized trials and observational studies), sample size, and outcome measures, making it difficult to draw firm conclusions. Thirdly, most studies focus on short-term outcomes (two to four weeks). Although this period is clinically relevant for the recommended short-term use of OXY, it limits understanding of long-term effects. Fourthly, the evidence mainly reports positive outcomes, with few studies showing neutral or negative results. Lastly, variability in patient populations (e.g., moderate-to-severe AR with nasal congestion) and treatment protocols across studies may limit the generalizability of findings. Therefore, well-designed clinical trials and real-world evidence studies are recommended to specifically assess the effectiveness and safety of this FDC for short-term use (≤14 days or ≤2 weeks) to further validate the review findings.

## Conclusions

The FF+OXY (27.5/50 µg) FDC nasal spray is a targeted approach for managing AR, particularly in patients with persistent nasal congestion. This FDC nasal spray, an intranasal decongestant, and an INCS address both decongestion and underlying inflammation in patients with AR, particularly when congestion significantly affects their QoL. This synergistic mechanism provides potent anti-inflammatory effects from FF, and OXY provides a rapid onset of action within 25 seconds, providing quick vasoconstrictive relief. The FDC nasal spray of FF+OXY (27.5/50 µg) was effective in improving TNSS, TOSS, and TSS without causing significant AEs, including RM. This metered-dose nasal spray is available in India for the management of AR, and the recommended regimen is two nasal sprays in each nostril once daily at night. Although clinical studies indicate that the FDC nasal spray of FF+OXY (27.5/50 µg) can be used for up to 28 days, real-world clinical practice often limits its use to short-term treatment (≤14 days or ≤2 weeks). However, multicenter clinical trials with large sample sizes are recommended to confirm the efficacy and safety of the suggested FDC, assess improvements in treatment outcomes, and evaluate the risk of RM in patients with moderate-to-severe AR.
